# Supplementary material for: Inflammatory factors and risk of lung adenocarcinoma: a Mendelian randomization study mediated by blood metabolites
Source: Front Endocrinol (Lausanne). 2024 Aug 27;15:1446863. doi: 10.3389/fendo.2024.1446863 (PMC11384989; doi:10.3389/fendo.2024.1446863)
Supplement: Supplementary file 1 [file DataSheet1.zip › supplementary files/S2/GCST90200731/sensitivity-analysis.pdf]

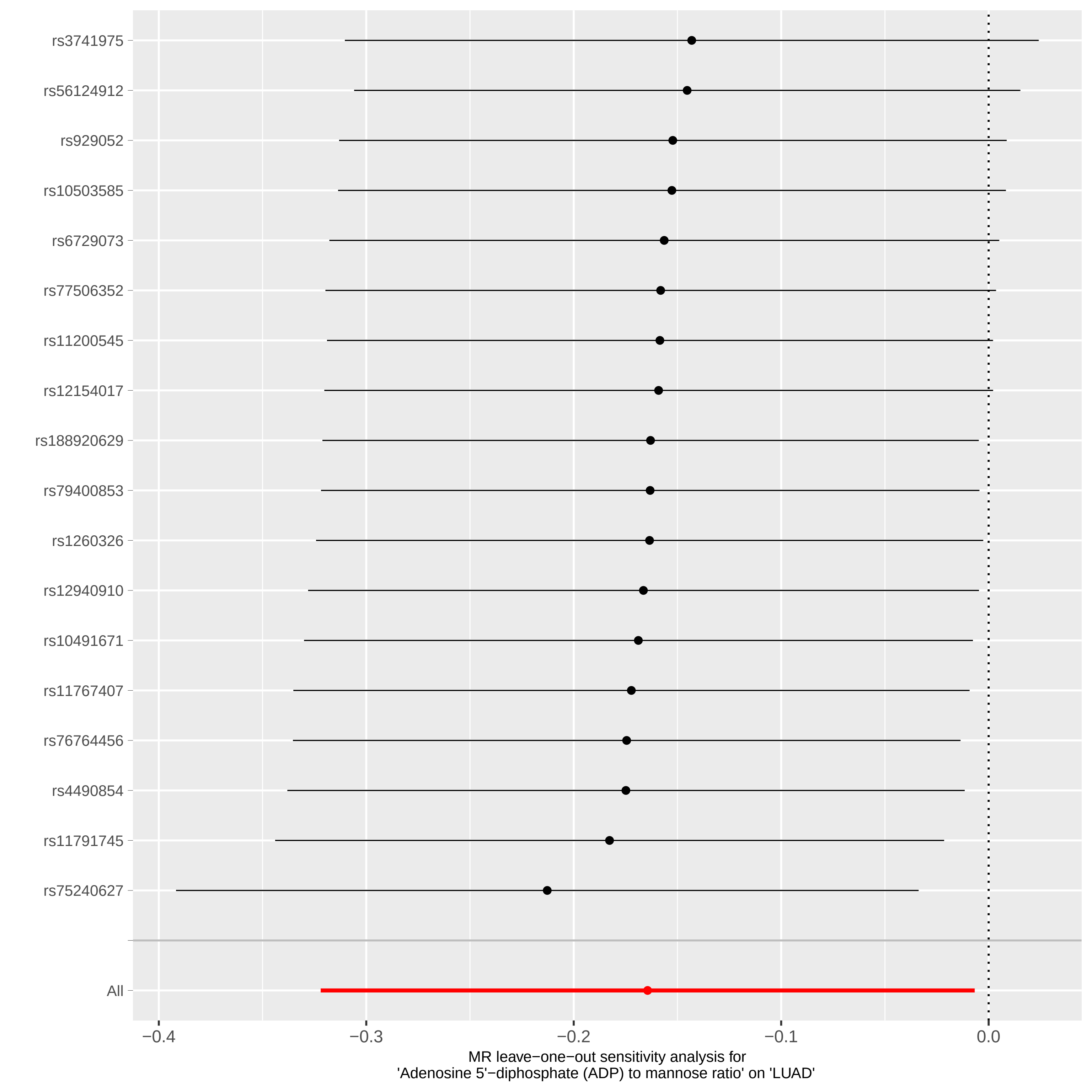
rs3741975
rs56124912
rs929052
rs10503585
rs6729073
rs77506352
rs11200545
rs12154017
rs188920629
rs79400853
rs1260326
rs12940910
rs10491671
rs11767407
rs76764456
rs4490854
rs11791745
rs75240627
All
−0.4
−0.3
−0.2
−0.1
0.0
MR leave−one−out sensitivity analysis for
'Adenosine 5'−diphosphate (ADP) to mannose ratio' on 'LUAD'
